# The Book World of Medicine and Science

**Published:** 1900-05-19

**Authors:** 


### The Book World of Medicine and Science.

### THREE BOOKS ON GYNÆCOLOGY.

Practical Manual of Diseases of Women and Uterine Therapeutics for Students and Practitioners. By H. Macnaughton-Jones, M.D., M.Ch. Eighth edition, revised and enlarged, with 640 Illustrations and 28 plates. (Baillière, Tindall, and Cox. 1900. Price 18s. net.)

Manuel Complet de Gynécologie Medicale et Chirurgicale. Par A. Lutaud. Nouvelle Edition, entièrement refondue, contenant la technique operatoire complete et 607 figures dans la texte. (Paris: A. Maloine. 1900.)

A Manual of Gynæcological Practice for Students and Practitioners. By Dr. A. Dührssen. Second English Edition, translated from the Sixth German Edition by John W. Taylor, F.R.C.S., and Frederick Edge, M.D.Lond., M.R.C.P., F.R.C.S. With 125 Illustrations. (London: H. K. Lewis. 1900. Price 6s.)

These three works are all of established reputation, and the number of editions they have run through proclaim their popularity. With so progressive an art as gynæcology, a book must of necessity either be revised at frequent intervals or die out of all memory, for the operations of ten years ago are in many cases obsolete, and the books describing them are thus not only useless but misleading to the student.

### Dr. Macnaughton-Jones.

Taking the new edition of Dr. Macnaughton Jones' work, for example, and referring to the article on the surgical treatment of uterine myomata, we see how necessary it is to retain a plastic mind and eschew all search for finality in this department of surgery. The last edition appeared only in 1897, yet, referring to it, the author now says that much that was then described has become "practically obsolete." He says: "I do not, therefore, occupy time in describing the extra-peritoneal treatment of the pedicle known as Hégar's method, or extra peritoneal-celio-hysterectomy. The temporary elastic ligature, the rope ligature of Tait, the clamping of the pedicle, the permanent elastic ligature, and the various plans for fixing and treating the pedicle, are interesting from the historical point of view, but have become obsolete as modern surgical methods." The same is said of a whole crowd of other methods, all of which, although they date from only a few years ago, are already dead and forgotten, so rapidly does time travel and fashion pass away in the gynæcological world.

The same thought is brought to mind if one recalls how large a place ovarian tumours and their treatment by ovariotomy at one time occupied in what was then the comparatively limited speciality of the gynæcologist, and if one then goes on to note how small a space in this book is taken up by the description of these diseases, and how thoroughly the centre of interest has of late years been shifted into other channels.

The criticism which we should still be inclined to make in regard to this book is much the same as that which we should have applied to former editions, namely, that notwithstanding the manner in which it has been kept in touch with modern progress, and however well the author describes the various procedures which are open to us in the management of the several diseases, he does not always indicate his own preferences, or show with sufficient precision in which cases he would himself advise that the various methods which he details should be employed. The book, however, is a most useful one as a work of reference, and is an admirable students' manual. Commencing with a description of the anatomy of the female organs and with the subject of case-taking, it then devotes some sixty pages to the "first steps of examination of a case," which strikes one as a somewhat ample allowance. Like the rest of the book this portion is very fully illustrated, which is well if only it does not lead the student to imagine that it is impossible to do gynæcology without all the appliances here depicted. Then comes a good deal about antiseptics, and this is a portion of the book which deserves much commendation, the precautions advised being the logical outcome of the acceptance of modern theories as to the microbic origin of septic diseases. After this we have a couple of chapters on some minor operations, and then comes the systematic description of the various diseases and their treatment. Perhaps it is worth mentioning, as showing how specialities tend to be limited only by the special aptitudes of those who practise them rather than by anatomical landmarks, that this work on gynæcology is now made to include affections of the rectum, of the bladder, the ureters, and even of the kidneys. Take it altogether, this edition is a vast improvement upon those that have gone before, and the work forms a very useful and instructive manual. The illustrations, which are scattered in great profusion throughout its pages, are of a very high order of merit, and both paper and printing are excellent.

### Dr. Lutaud.

Dr. Lutaud's work in its size and general arrangement corresponds very much with that by Dr. Macnaughton-Jones, and like it also is well printed and well illustrated. A little less emphasis is perhaps placed upon the methods necessary for obtaining asepsis, but it is clear that the object aimed at by both men is the same. Like Macnaughton-Jones, Lutaud also describes many operations, some of which he no doubt would not practise, and it must be added that he describes some of those proceedings which Macnaughton-Jones stigmatises as "obsolete," leaving his readers to take their choice, which is perhaps unfortunate. On the other hand, in his clearness of description Lutaud is admirable. The French method of tabulation and of describing operations in numbered stages, although not perhaps exactly true to life, and not always intended to be slavishly followed in practice, is one which lends itself admirably to description, and no one can doubt the clarity of M. Lutaud's account of the various operations which he describes. The book will be found very useful by learners and as a work of reference.

### Dr. Dührssen.

Dührssen's book is a much smaller one than either of those we have so far mentioned, but it contains an enormous amount of information. The descriptions are good although they are short, and the practice recommended is thoroughly modern, and evidently the outcome of much experience. As a small and handy manual it has a great deal to recommend it, and the insertion of a chapter on the anatomy of the parts in this edition has rendered it much more complete than it was before. Naturally, there is a good deal about vaginal cœliotomy, an operation which Dr. Dührssen has practised very largely. There is no doubt that the vaginal route is available in many cases, and where the disease is accessible from this direction, the operation described by the author is a most admirable one. How far it is desirable to go in pushing this route for the removal of myomata, except when quite small, is another question. The book will be found very useful by a large class of readers. It contains a wonderful amount of information considering its size, but, perhaps in consequence of its condensation, it requires somewhat careful reading. As a guide in case of doubt it is excellent in that the advice given is not merely sound but definite, which is a great gain. Some of the illustrations are good, others are distressingly "fogged," to use a photographic term.

